# Unusual Delayed FDG-PET/CT Hypermetabolism Due to Charcoal-Induced Granuloma

**DOI:** 10.5334/jbsr.1549

**Published:** 2018-04-20

**Authors:** Quentin Rahier, Fabrice C. Deprez

**Affiliations:** 1 CHU UCL Namur, BE

**Keywords:** Charcoal induced granuloma, FDG-PET-CT false positive

## Case

**Figure 1:**
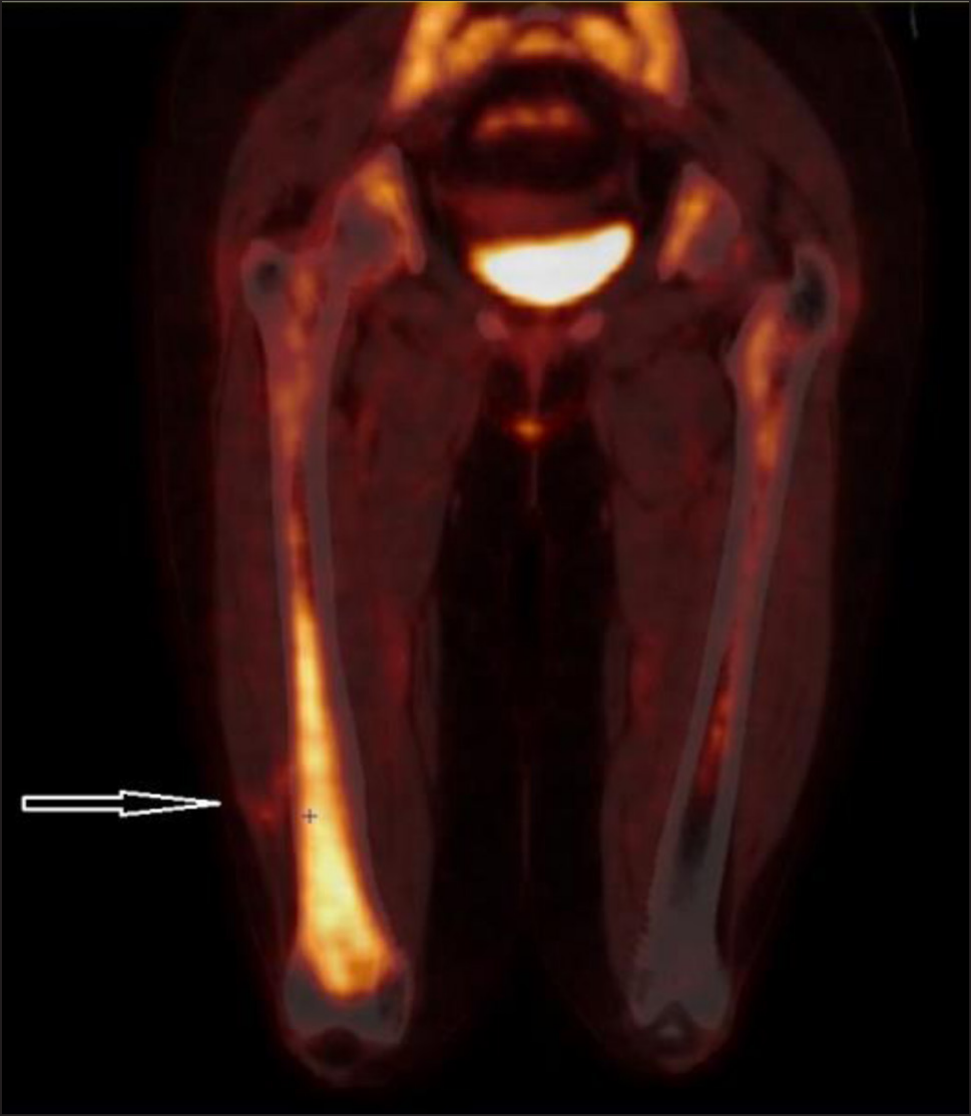


**Figure 2:**
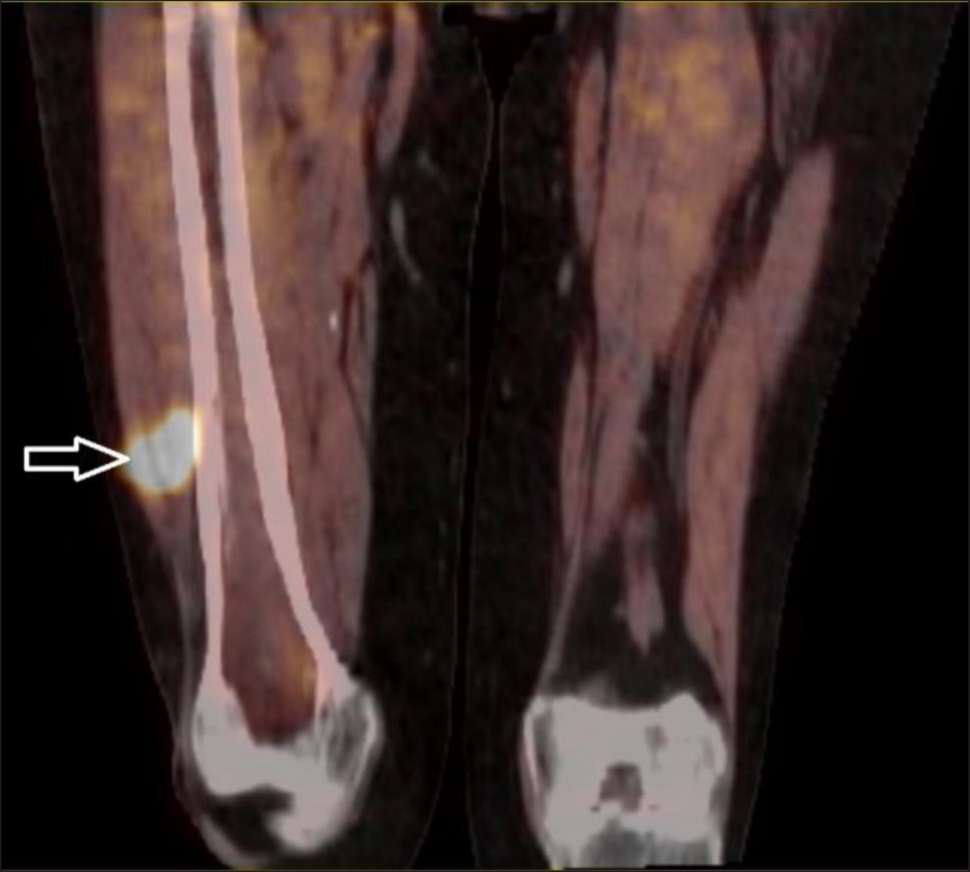


**Figure 3:**
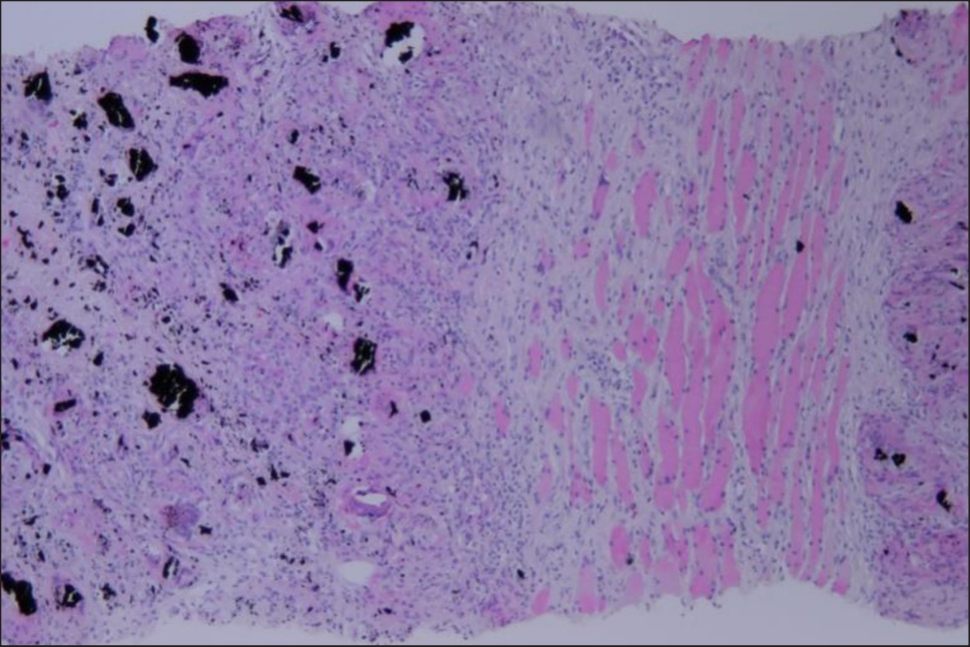


A 63-year-old patient initially presented with right leg pain. First imaging studies (not shown) revealed a tumoral infiltration of the distal half of the femur. As sarcoma was initially suspected, a percutaneous biopsy was performed with charcoal (black carbon) deposit along needle tract. It revealed a high-grade follicular lymphoma and the patient was treated with 8 cycles of R-CHOP. Four months after treatment initiation, first follow-up ^18^F fluorodeoxyglucose (FDG) Positron Emission Tomography coupled with Computed Tomography (PET/CT) showed a persistent strong FDG uptake in the femur along with the biopsy track (Figure 1, arrow). Six months later, a follow-up FDG-PET/CT after completion of treatment showed a complete femoral metabolic response, but appearance of a hypermetabolic lesion (SUV_max_ = 17.3) in the muscle tissues next to the previous lymphoma localization (Figure 2: PET/CT – arrow: biopsy track). US-guided biopsy was performed and showed dark-pigmented fragments. Microscopically, striated muscle tissues and subcutaneous tissues with granulomatous inflammation that comprised multinucleated giant cells, fibrosis and charcoal deposits were observed (Figure 3: H-E coloration, ×10).

## Comment

Charcoal, also called black carbon, is used for preoperative localization of tumor (mainly for breast tumors) or needle track localization after biopsy (mainly sarcomas biopsy, due to a potential risk of tumor seeding along needle track with this type of tumor).

Foreign-body granulomas can lead to false-positive findings of FDG PET/CT due to strong FGD uptake. On unenhanced CT, granulomas can show variable configurations, as delimited oval or round shape, or amorphous and linear configuration. Charcoal-induced granulomas usually show a spontaneous high attenuation, due to the high density of carbon particles, and no remarkable enhancement after contrast injection. On US studies, charcoal granulomas are characterized as irregular-shaped lesions, most often with hyperechogenicity and posterior shadowing. On the contrary, tumor recurrences often appear hypoechoic.

As described by Jin Woo Choi, charcoal is known to be a safe and biologically inert material when injected subcutaneously and may remain in situ for up to 60 days without triggering any inflammatory reaction [1]. However, at longer term, charcoal particles will be ingested and slowly degraded by macrophages, inducing progressive inflammation reaction and, if it remains in situ for more than six months, substantial foreign body granulomas. Consequently, FDG-PET hyperintensity due to charcoal-induced granuloma is very rarely observed, because initial FDG-PET/CT performed just after charcoal subcutaneous injection does not show any early inflammatory reaction, and most often tumor and needle tract are surgically removed before later PET-CT follow-up. As our patient was only treated by chemotherapy, we could observe this rare situation that could have been misinterpreted as recurrent tumor.
